# A cryptic host–parasitoid interaction reduces the impact of heatwaves on *Drosophila* host populations

**DOI:** 10.1098/rspb.2025.1527

**Published:** 2025-11-26

**Authors:** Jinlin Chen, Owen T. Lewis

**Affiliations:** ^1^ School of Life Sciences, Nanjing University, Nanjing, Jiangsu, People’s Republic of China; ^2^ Department of Biology, University of Oxford, Oxford, UK

**Keywords:** thermal tolerance, parasitism resistance, nutrition, cross-protection, multiple stressors

## Abstract

Laboratory measures of thermal tolerance are used to predict population responses to climate extremes, but rarely account for co-occurring biotic stressors associated with consumers and resources. Among consumers, parasitoids have especially intimate interactions with hosts that likely both depend on and alter host physiology. However, the context-dependent interplay between host reactions to parasitism and heat remains understudied. We applied a factorial design of heatwave, parasitism and nutrition treatments on three rainforest *Drosophila* species to test whether parasitoid infection reduces host heat tolerance, particularly under nutritional deficiency. We found that high-yeast diets increased the resistance of *Drosophila* to parasitoids but decreased their survival during heatwaves. Surprisingly, exposure to parasitoids reduced the susceptibility of host populations to heatwaves compared to null models accounting for combined mortality effects; this reduction was observed under yeast-rich diets and independent of host susceptibility to their native parasitoids. We reveal that parasitoids exert cryptic effects on hosts they cannot successfully develop within, with positive fitness consequences for hosts under extreme heat. This consistent positive interactive effect across native hosts suggests a general crosstalk between physiological pathways for immunity and heat tolerance—a critical consideration for predicting population-dynamic responses to climate change within community networks of closely interacting species.

## 1. Introduction

As record-breaking heatwaves occur with increasing frequency around the globe [1–3], many species are now experiencing more extended and frequent exposure to stressful temperatures [4,5], leading to unusual fluctuations in population sizes that could cause significant disruption to their ecological functions [6–9]. Population dynamics are also heavily regulated by biotic interactions within ecological communities. Whether biotic interactions tend to exacerbate the impact of the changing climate [10,11] or stabilize populations in the face of abiotic perturbations [12–14] is a critical but unanswered question. While density-mediated indirect effects could be quantified by concurrently considering the thermal profiles of interacting species, the joint effects of biotic and abiotic stressors are often non-additive and hard to predict [15–17]. Understanding the alteration of physiological states by biotic interactions and the crosstalk within the physiological networks is key to making robust and generalizable predictions [15].

Parasitoids are insects whose larvae develop inside or on the bodies of other arthropods, killing the host during their own development [18], often with marked impacts on host population dynamics [19]. Although the ecological function of parasitoids in suppressing host populations has often been equated to predation [20], host–parasitoid interactions entail more intimate and intricate processes including host immunity and immune evasion by parasitoids [19,21]. Larval parasitoids, in particular, have to co-live with their hosts until the hosts pupate, evading the host’s immune system while not killing the hosts as larvae. A parasitoid infection yields three possible outcomes: the host survives while the parasitoid fails to develop, death of both organisms due to the infection [19] or parasitoid emergence at the expense of the host. We define parasitism success as the probability of the third outcome, and host susceptibility as the loss of hosts from the last two outcomes combined. In natural communities, different host species exhibit variations in the relative frequencies of these three outcomes.

The tight physical association of parasitoids with their hosts throughout development is likely to affect the ability of parasitoid-carrying hosts to withstand other stressors [22], paralleling the effects of parasites and pathogens on thermal tolerance [23]. Differences in hosts’ heat tolerance have been suggested to influence how heat alters the outcome of parasitism [16]; likewise, host species with varying capabilities to mount an immune response to parasitism may differ in the consequences of parasitism for heat tolerance. This non-reproductive effect of parasitoid infection, and its species dependence, has seldom been studied [24].

The requirement for nutrients is a fundamental factor linking an organism’s resistance to different abiotic and biotic pressures. Conflict in resource allocation has been suggested as the driver for physiological trade-offs between resistance traits, as both processes rely on energy and raw materials to activate molecular pathways [25–28]. Thus, the limitation on critical nutrients, such as amino acids provided by yeast, will likely impair responses to stressors (see [29] for parasitoid resistance and [30] for cold tolerance) and is the prerequisite for negative interactive effects of multiple stressors to play out [31]. On the other hand, shared defence pathways may lead to positive interactive effects (cross-protection, as documented by [24,32]). For example, heat shock proteins can be elevated by various abiotic and biotic stressors [33,34], offering protection from subsequent stress, including extreme temperatures [35]. Such cross-protection between immune and thermal responses has been documented in various insect systems [36,37]. This positive interactive effect is more likely to be pronounced when resources are abundant, as more building blocks for inducible stress responses are available.

**Figure 1.**
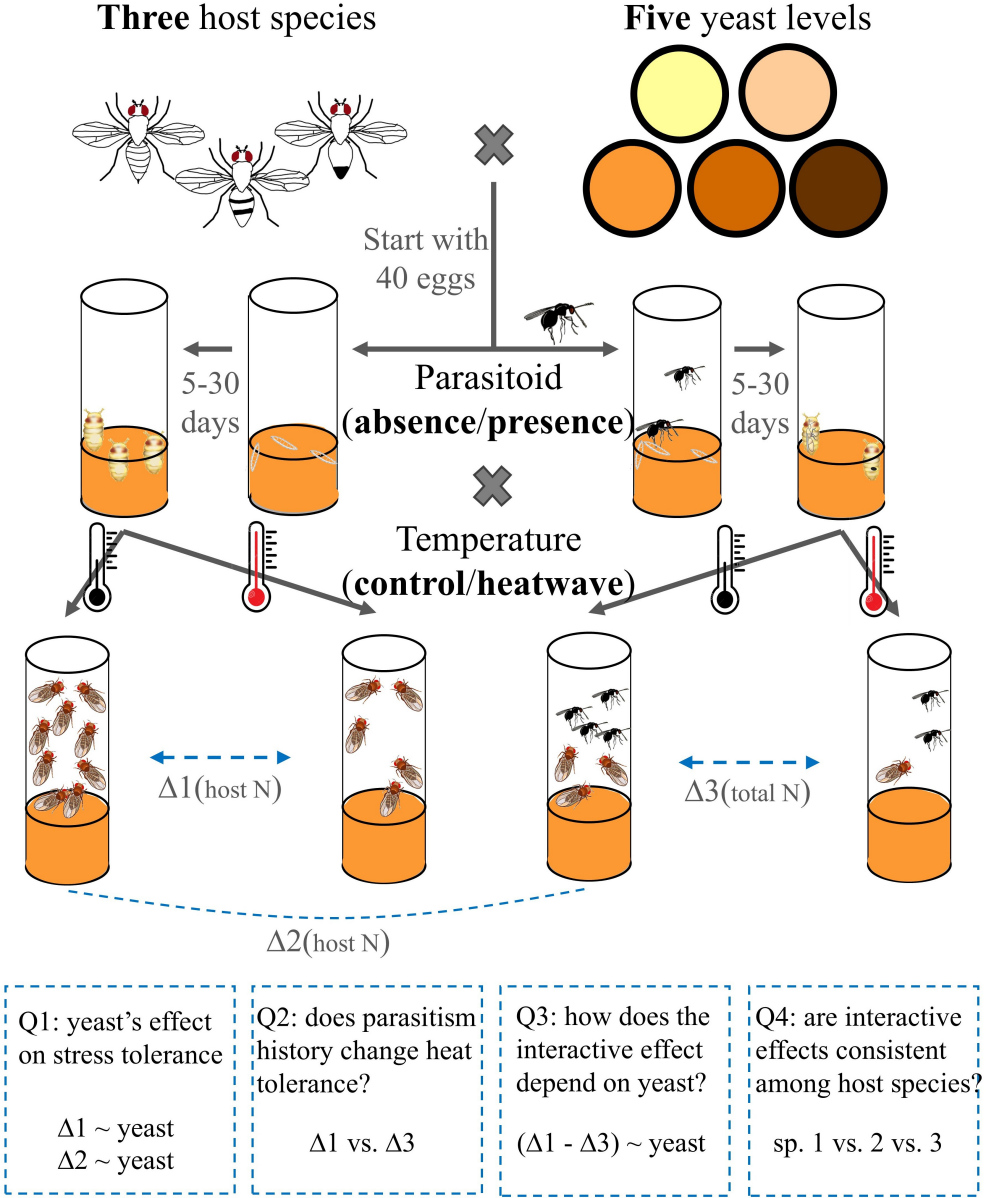
Research framework and experimental procedures. For each of the three species, we devised a factorial design of five diet yeast levels, two parasitism treatments and two temperature treatments, totalling 20 treatments (*n* = 6). Each experimental vial started with 40 fly eggs. Vials were randomly assigned to the parasitoid and non-parasitoid treatments. Once the larvae started to pupate (diet and parasitoid treatments influence development time), vials were randomly assigned to either the heatwave (red thermometer) or the control (black) treatment for 1 day. Vials were then kept at the control temperature, and adult hosts and parasitoids were counted. ∆ represents the difference in adult numbers observed between vials of different treatments. At the bottom, four research questions and their relevant data and tests are specified.

**Figure 2.**
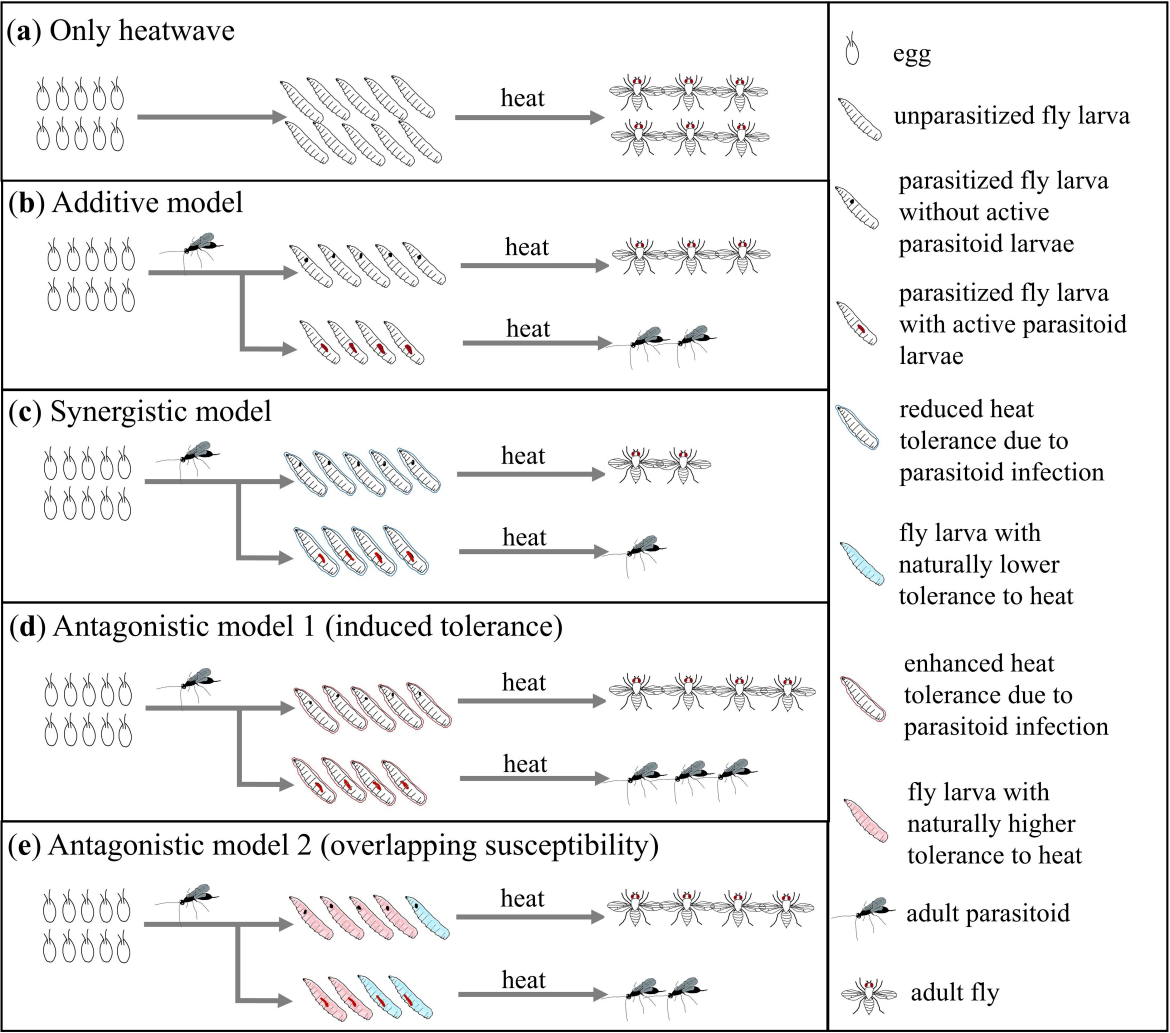
Models for different types of interactive effects between parasitism and heatwaves on the survival of hosts. (a) The sole effect of a heatwave. (b) The additive model is the null model, with parasitism and heatwave each independently causing a certain level of host mortality. (c) In the synergistic model, hosts with experience of parasitoid infection are more likely to die from heatwaves than unparasitized hosts. (d) In the first antagonistic model (induced tolerance), parasitoid infection induces higher tolerance to heatwaves among fly larvae. (e) The second antagonistic model suggests that hosts with a naturally higher tolerance to heatwaves are more likely to evade the parasitoid infection. For simplicity, the death rate during host development without parasitism and heatwave stress is not illustrated; this death rate was consistent overall, but higher only if on yeast-free diets.

Here, we use three heat-sensitive tropical rainforest *Drosophila* species and a co-occurring parasitoid species to investigate whether interactions with parasitoids alter the tolerance of host populations to extreme temperatures, and how this impact of parasitism is moderated by resource availability and host identity. Figure 1 summarizes the experimental design and research questions to be tested. We first established the relationships between nutrient (yeast) abundance and the hosts’ ability to survive either a heatwave or exposure to parasitoids. Second, we tested the hypothesis that infection by parasitoids would lower the tolerance of hosts to heatwaves (synergistic model in figure 2c), because of the cost of repairing damage or mounting an immune response. If yeast is shown to be a critical nutrient for both immune defence and heat tolerance, we further predicted that scarcity of protein in the diet would increase the negative impact of parasitism on heat tolerance. Lastly, we carefully selected native hosts with varying susceptibility to the parasitoid species to investigate the generality of the interactive effects between parasitism and heatwaves.

## 2. Methods

### (a) Overview of study system

**Figure 3.**
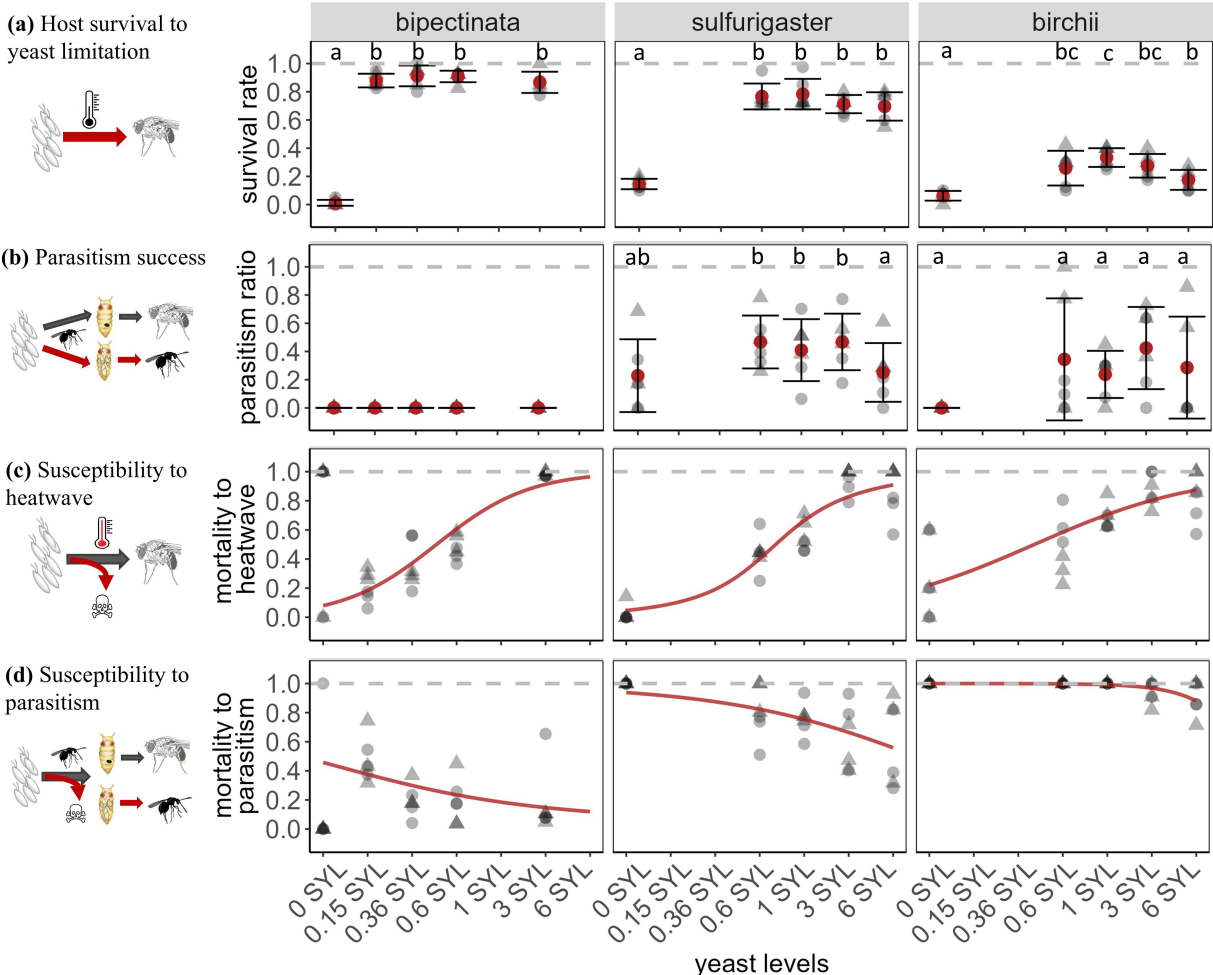
The effect of dietary yeast on host and parasitoid performance. The diagrams in the left column indicate the processes (red arrows) contributing to each measurement. (a) Host survival without heatwave and parasitism. (b) Parasitism success measures the ratio between the number of parasitoids and the average number of available hosts on the corresponding yeast levels (hosts that could have completed development without exposure to parasitoids). (c) Susceptibility to heatwave measures death caused by the heatwave treatment. (d) Susceptibility to parasitism, including the mortality of fly larvae as a result of parasitoid attack as well as hosts that were successfully parasitized. The *x*-axis is the dietary yeast level relative to the usual food (1 standard yeast level (SYL)). Grey points are observations (*n* = 6); darker colours indicate overlap of points. Blocks are indicated by data point symbols. In (a) and (b), the red point is the mean at each yeast level, and the error bar indicates ±1 s.d.; letters above the data columns indicate statistically different groups as determined by *post hoc* pairwise comparisons. Curves in (c) and (d) are the generalized linear model estimates of susceptibilities in response to yeast levels, and the red colour represents the significant coefficient for yeast.

We conducted the experiments using laboratory cultures of one parasitoid wasp species (*Asobara* sp., lab strain KHB [38], subsequently referred to as ‘*Asobara’*) and three *Drosophila* species. The laboratory cultures were established within 5 years of the experiment from natural tropical rainforest communities in Queensland, Australia. *Asobara* attacks second-instar (of three total instar stages) *Drosophila* larvae, with a single offspring emerging from each host pupa. The ‘native hosts’, *D. bipectinata*, *D. sulfurigaster* and *D. birchii*, co-occur naturally (along with *Asobara*) in the Australian wet tropics [39] and were chosen for our experiments because of their differing susceptibilities to *Asobara* (figure 3c). We additionally included a laboratory strain of the species *D. melanogaster* (Dahomey strain maintained in mass population; referred to as the ‘non-native host’), which does not co-occur naturally with *Asobara* [39] and is fully susceptible to it (electronic supplementary material, figure S1). The *D. melanogaster–Asobara* pair was investigated in parallel with all native hosts to monitor parasitoid performance, as well as under a more severe heatwave scenario specific to *D. melanogaster*.

Prior to the experiment, parasitoids were maintained as an isofemale line for approximately 80 generations in the laboratory, using the novel and fully susceptible host *D. melanogaster* to minimize adaptation to any specific host species. Isofemale lines (collected in different mountain ranges and elevations) of rainforest *Drosophila* species had been cultured for about 30 generations before being used to construct mass-bred lines [40] and then maintained in mass population cages for 2–3 years under controlled temperatures (initially at 25°C, and then at 23°C from March 2020 to reduce maintenance frequency). Rainforest *Drosophila* were maintained on standard fly food (20 g saf-levure dry yeast (*Saccharomyces cerevisiae*), 48 g NATCO cornflour, 6 g bacterial-grade agar, 30 g commercial sucrose and 10 ml of 10% nipagin solution in 600 ml of boiling water). This is referred to as the standard yeast level (SYL) for our experiment.

### (b) Experimental design

Overall procedures are summarized in figure 1. We reared 40 fly larvae on five levels of yeast and at the same time subjected them to parasitism treatments (with or without *Asobara*). Upon pupation, any parasitoids were taken out, and flies were subjected to heatwave treatment (control versus a 24 h heatwave, detailed below). Host or parasitoid adults that successfully emerged from each vial were recorded. For each native host–parasitoid pair, we investigated all combinations of treatments (5 × 2 × 2 = 20) with six replicate vials of each combination (split equally into two blocks and conducted 1 day apart). For *D. melanogaster*, we also used six replicates when investigated in parallel with the native hosts, and nine replicates when investigated under its specific heatwave level.

*Drosophila* adults were reared at low density from eggs obtained from stock before harvesting their eggs to start the experiment. For each species, eggs were harvested from two to three cylindrical containers housing 100 mixed-sex adults (200 for *D. birchii* because their fecundity is generally lower). Eggs laid on the yeast paste at the bottom of containers were washed and well-mixed before being picked into experimental vials.

Forty *Drosophila* eggs were transferred onto the surface of the fly food in each vial (diameter × height = 25 × 95 mm). We modified the nutritional values of *Drosophila* food by adding a varying amount of dried yeast, a primary source of protein [41], while keeping other contents unchanged. Levels of yeast are expressed in comparison to the yeast level in the standard food used for stock: 0% SYL, 15% SYL, 36% SYL, 60% SYL, 100% SYL, 300% SYL, 600% SYL. These levels were used in previous studies that revealed the critical roles of yeast in influencing *Drosophila* fitness, such as immune responses and tolerance to abiotic stress [28,30,41–43]. Different sets of five out of the seven abovementioned levels were selected for different species based on pilot studies on their parasitism resistance. For example, the five yeast levels used for *D. bipectinata* were lower overall than for other species, to increase variation in susceptibility to parasitoids.

Immediately after transferring eggs, two female and two male *Asobara* wasps were introduced into half of the vials at each yeast level. Experimental vials were then kept at the control temperature regime (22°C for 4 h (light), 26°C for 4 h (light), 22°C for 4 h (light), 18°C for 12 h (dark)). Vials were checked daily, and any adult parasitoids that died were replaced. Once the fly larvae in at least half of the replicates from a particular treatment combination started pupating, parasitoids were removed from that group. Diet affects the speed of larval growth, changing the duration of the window when larvae are vulnerable to parasitism. Our design exposed the entire larval stage of hosts to parasitoids across all dietary treatments. We conducted the same procedure for the *D. melanogaster–Asobara* pair to confirm that parasitoids were able to parasitize all 40 hosts within the exposure time (electronic supplementary material, figure S1).

Flies with or without exposure history to parasitoids were then subjected to temperature treatments. We applied temperature treatment after removing parasitoids so that the direct impact of heatwaves on the performance of adult parasitoids was excluded. As life stage influences heat tolerance [44], we consistently applied temperature treatments to hosts around their larva-to-pupa transformation. The heatwave dates varied because diet and the presence of parasitoids affected larval growth. Once pupation started (coinciding with the removal of parasitoids), half of the vials were randomly selected to experience a 1 day heatwave, while the other half were handled in the same way but immediately returned to the control temperature. The magnitude of the heatwave varied among species, based on heat tolerances tested in pilot experiments (*D. birchii* and *D. sulfurigaster*: 30°C for 4 h (light), 34°C for 4 h (light), 30°C for 4 h (light), 24°C for 12 h (dark); *D. bipectinata*: 30°C for 4 h (light), 38°C for 4 h (light), 30°C for 4 h (light), 24°C for 12 h (dark)). Vials were returned to the control temperature after the heatwave treatment, and adult *Drosophila* and parasitoids reared from each vial were counted.

We tested the *D. melanogaster–Asobara* pair under the abovementioned heatwave treatments designed for the native hosts. Since *D. melanogaster* is fully susceptible to *Asobara*, this allowed us to assess the direct effect of heatwave treatments on parasitoid survival, independent of effects of heatwave conditions on host immunity. Additionally, *D. melanogaster* was investigated under a heatwave regime that reflects their higher tolerance of heat. Due to equipment constraints, we used a constant 25°C for the control regime, and we applied a 6 h constant heatwave to the *D. melanogaster* by quickly moving them from 25°C control temperature room to an incubator set at 37°C, 3 h after their daylight started. More details are provided in the electronic supplementary material, S2.

### (c) Data analysis

#### (i) Effects of dietary yeast on survival, heatwave tolerance and susceptibility to parasitoids

We calculated several measures of host and parasitoid performance based on numbers of eggs, counts of emerged *Drosophila* and emerged parasitoids to examine the influence of dietary yeast:

Host survival (without heatwave and parasitism) $=\frac{{\text{N}}_{\text{host}}}{{\text{N}}_{\text{egg}}}$

Parasitism success (without heatwave) $=\frac{{\text{N}}_{\text{parasitoid}}}{{\text{N}}_{\text{host average in control}}}$

Host susceptibility to heatwave (without parasitism) $=1-\frac{{\text{N}}_{\text{host in heatwave treatment}}}{{\text{N}}_{\text{host average in control}}}$

Host susceptibility to parasitoid (without heatwave)$=1-\frac{{\text{N}}_{\text{host in parasitoid treatment}}}{{\text{N}}_{\text{host average in control}}}$

*N*_host average in control_ is the average number of hosts that could survive to adulthood on the corresponding yeast level without heatwave and parasitism. As survival is influenced by yeast levels, this mortality is excluded from calculating the following three rates by comparing the observed emergence with *N*_host average in control_. Parasitism success measures the proportion of available hosts (those that could have survived in the absence of parasitoids) being successfully converted to adult parasitoids (figure 3b). Host susceptibility to heatwave quantifies the additional death caused by the heatwave treatment (figure 3c). Likewise, host susceptibility to parasitoids quantifies the additional death of hosts in comparison with parasitoid-free controls (figure 3d).

In yeast-free vials, host survivals were minimal (figure 3a), and the small number of survivors led to high uncertainty when using them as the denominator to calculate mortality rates. From 540 vials, nine and five observations from yeast-free vials showed a higher number of hosts in heatwave or parasitism treatments, respectively, than *N*_host average in control_. As mortalities cannot be negative, we adjusted them to 0 following Pardikes *et al*. [45]. In one vial of *D. birchii* at 0.6 SYL where more wasps emerged than *N*_host average in control_, we set parasitism success to 1. All rate measures were modelled using generalized linear models assuming a binomial error distribution (link function ‘logit’):

‘Host survival’ or ‘parasitism success’ ~ yeast levels + block

‘Host susceptibility to heatwave’ or ‘host susceptibility to parasitoid’ ~ rank (yeast levels) + block

The first two response variables did not respond linearly to yeast levels. Therefore, *yeast level* was modelled as a categorical variable, followed by *post hoc* pairwise comparisons using the *emmeans* package [46]. To study the monotonic relationship between *yeast level* and susceptibility to either heatwave or parasitoid, we assigned ranks to *yeast levels* from 1 (low yeast content) to 7 (high yeast content) and then modelled the susceptibility as a function of the rank of *yeast level* and *block*. For the susceptibility of *D. sulfurigaster* to heatwave, yeast rank and block had significant interactive effects; we included the interaction term in this model.

### (d) Interactive effects between parasitism, heatwave and nutritional treatments

We then investigated whether past exposure to parasitoids generally influenced host survival to heatwaves and how such interactive effects are modulated by yeast. We fitted a linear regression model across all host species with a three-way interaction. The response variable was log-transformed total number of emergence (hosts + parasitoids), added with 1 to avoid log(0) values. Note that adding ‘0.1’ or ‘2’ did not change the sign and significance of the relevant coefficients but altered the effect sizes of factors. Therefore, absolute effect sizes derived from the ‘+1’ model should be interpreted with caution. The log transformation ensures that the interactive term measures the true synergistic or antagonistic effect between two stressors on survival [47]. We used simulations (electronic supplementary material, S3) to demonstrate that false-positive rates of detecting a parasitism × heatwave interaction constantly remain low as overall susceptibilities change significantly with yeast levels, thus excluding statistical artefacts of the three-way interactions.

We modelled the transformed total emergence as a function of *heatwave*, *parasitism*, *yeast level* (ranked and centred), all two-way and three-way interactions among these treatments, *species ID* and *block,* using the *brms* package [48]. *Yeast levels* were ranked as described before and then centred around the 100% SYL to avoid multicollinearity issues when testing the interactive effects of yeast levels with other factors. To further investigate the ultimate outcomes for host populations, we fitted the same model but with log-transformed adult host number as the response variable (excluding the *D. birchii*–parasitoid pair because most *D. birchii* had died from parasitism). We fitted additional models for each *Drosophila*–parasitoid pair separately, using *heatwave*, *parasitism*, *yeast level* (as a categorical variable), all two-way and three-way interactions, and *block* as predictors. Analyses were performed in R v. 4.3.0 [49]. The data and code for analysis that support the findings of this study can be viewed on figshare at https://doi.org/10.6084/m9.figshare.29605136.v1.

## 3. Results

### (a) Effects of dietary yeast on survival, heatwave tolerance and susceptibility to parasitoids

The yeast-free treatment significantly decreased egg-to-adult survival rates for all *Drosophila* species (figure 3a). A small amount of yeast added to the food significantly enhanced survival, which then remained steady as yeast content increased further. Parasitism success varied little across yeast levels and was only significantly lower in one case (yeast level 6 SYL for *D. sulfurigaster*; figure 3b). In the absence of parasitoids, higher dietary yeast increased the susceptibility to heatwaves of all *Drosophila* species (figure 3c; table 1). In contrast, higher diet yeast decreased host susceptibility to parasitoids (figure 3d; table 1). The experimental block had significant coefficients for *D. sulfurigaster* for both susceptibilities and a positive interactive effect with yeast levels only for *D. sulfurigaster*, driven by a higher number of emergences in the latter block. Although the reason for this difference is unknown, relationships between yeast levels and susceptibility to heat were positive for both blocks. Changes in host susceptibility did not always correspond to parasitism success; e.g. the presence of parasitoids caused significant mortality of *D. bipectinata* (mean mortality across nutrition treatments = 24%), even though no adult parasitoids appeared to complete development on this *Drosophila* species (figure 3b). The effects of yeast levels on susceptibility to either heatwave or parasitoids were similar for native and non-native *Drosophila* (electronic supplementary material, S2, figure S4).

**Table 1.** Statistical analysis of the effect of dietary yeast on the response of hosts to either heatwave or parasitism. Susceptibilities were modelled assuming binomial error distributions.

species	predictors	susceptibility to heatwave		susceptibility to parasitoids	
		coefficient	***p*-value**	coefficient	***p*-value**
*D. bipectinata*	intercept	−3.51	<0.0001	0.16	0.46
	yeast	0.96	<0.0001	−0.32	<0.0001
	block	0.20	0.21	−0.10	0.52
*D. sulfurigaster*	intercept	−3.69	<0.0001	3.09	<0.0001
	yeast	0.66	<0.0001	−0.43	<0.0001
	block	−3.69	0.0002	0.36	0.025
	block : yeast	0.91	<0.0001	—	—
*D. birchii*	intercept	−1.69	0.001	12.00	0.0002
	yeast	0.53	<0.0001	−1.39	0.005
	block	−0.21	0.44	−0.56	0.46

### (b) Interactive effects between parasitism, heatwave and nutritional treatments

According to the regression model for the three native host *Drosophila* species (coefficients and their confidence intervals are shown in table 2, *R*^2^ = 0.47), at the reference yeast level (1 SYL), heatwave treatment alone significantly decreased the total number of hosts and parasitoids by 75%. Parasitoid treatment alone significantly decreased total emergence by 46%. Higher yeast increases the mortality caused by heatwave (a significant negative two-way coefficient), consistent with the previous finding when analysing the effect of yeast alone. When parasitoids were present, the total mortality caused by the heatwave was significantly reduced to 50% (interaction coefficient of heatwave and parasitism = 0.68). The three-way interaction between parasitism, heatwave and yeast treatments was significantly positive, meaning that the positive interactive effect between parasitism and heatwave treatments grew stronger when yeast levels were higher. The number of adult flies that emerged followed the same pattern as the total emergence (table 2, *R*^2^ = 0.41): the presence of parasitoids significantly reduced host mortality by heatwave from 80% to 63% (a significant positive two-way interaction), and this antagonistic effect tended to be stronger (but not significantly so) with higher yeast levels.

**Table 2.** Regression model for native hosts to examine the interactive effects among heatwave, parasitism and yeast treatments on total emergence (log-transformed) and adult host numbers (log-transformed). CI stands for credible interval.

	total emergence		adult host number	
	coefficient	95% CI	coefficient	95% CI
intercept	3.51	[3.24 to 3.78]	3.93	[3.59 to 4.29]
heatwave	−1.37	[−1.66 to −1.08]	−1.59	[−2.01 to −1.19]
parasitism	−0.62	[−0.90 to −0.33]	−0.89	[−1.29 to −0.49]
yeast	0.29	[0.20 to 0.38]	0.39	[0.25 to 0.52]
host ID: *D. sulfurigaster*	−0.30	[−0.54 to −0.05]	−0.81	[−1.09 to −0.54]
host ID: *D. birchii*	−1.40	[−1.64 to −1.16]	—	—
block	0.07	[−0.12 to 0.26]	−0.15	[−0.41 to 0.10]
parasitism × heatwave	0.68	[0.27 to 1.10]	0.59	[0.02 to 1.16]
parasitism × yeast	0	[−0.13 to 0.13]	−0.05	[−0.23, 0.13]
heatwave × yeast	−0.41	[−0.54 to −0.28]	−0.50	[−0.69, −0.32]
parasitism × heatwave × yeast	0.23	[0.05 to 0.41]	0.24	[−0.02, 0.50]

**Figure 4.**
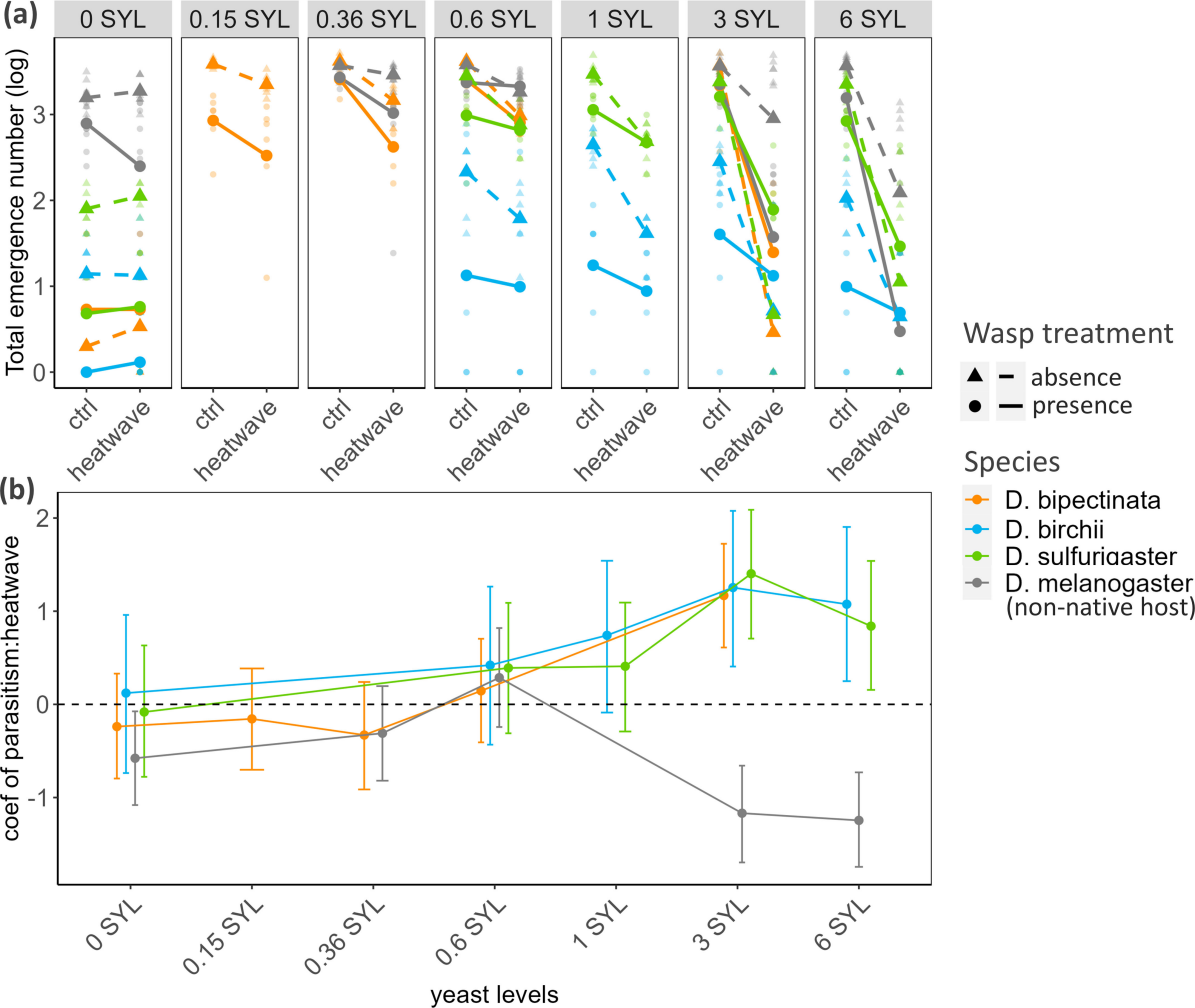
Interactive effects of parasitism and heatwave treatments. (a) The heatwave’s effects on the total emergence of adult flies and parasitoids, with or without parasitism treatment. Four species are plotted in different colours. Triangular points and dashed lines represent means for samples without exposure to parasitoids prior to the heatwave, while round points and solid lines represent means for samples with parasitism treatment. Underlying data are shown in translucent colours corresponding to their treatments. (b) Yeast levels modulate the interactive effects of parasitism and heatwave treatments. Medians and 95% credible intervals (error bars) of posterior estimates for the interactive terms are plotted against yeast levels. The grey dashed line indicates no significant interaction.

For each native host–parasitoid pair at high-yeast levels, prior exposure to parasitoids weakened the negative impact of heatwaves on total emergence, illustrated by the shallower slopes and significantly positive interaction coefficients in figure 4. In contrast to the three native hosts, when subjected to a more severe heatwave scenario specific to *D. melanogaster*, the *Asobara–D. melanogaster* pair showed a negative interactive effect between heatwave and parasitism when yeast levels were high (figure 4).

## 4. Discussion

Contrary to our hypothesized synergistic model (figure 2c) where parasitism makes post-exposure hosts more vulnerable to heat, exposure to parasitoids reduced the negative impact of heatwaves on host populations, even for a host species, *D. bipectinata*, that was not a suitable host for the focal parasitoid species (i.e. where no successful parasitism occurred). This interactive effect was stronger when yeast levels were high, which corresponded to low susceptibility to parasitoids and high susceptibility to heatwaves. Two-way and three-way interactive effects between heatwaves, resource quality and parasitism have similarly been found in a study of parasitoid fitness and life-history traits of a Lepidoptera host [17]. Both studies draw attention to the context dependence of thermal sensitivity: the legacy of biotic interactions influences heat tolerance, and such an impact also depends on resource quality.

### (a) Effects of nutrition on susceptibility to a single stressor

Our findings align with research indicating dietary protein’s critical role in host immunity across taxa, including *Drosophila* [28,29,50,51]. However, the higher heat tolerance observed under low yeast conditions contradicts our initial hypothesis and highlights the need to differentiate food scarcity from a lack of particular nutritional components. While starvation has been found to consistently reduce heat tolerance [52–54], studies on protein-to-carbohydrate ratios produced mixed results [55]. Le Rohellec & Le Bourg [56] similarly found that individual female adults survived heat longer when reared on a diet without yeast supplement. A high-carbohydrate and low-protein diet has been suggested to increase blood trehalose levels [55,57], a key stress-response chemical linked to improved thermal tolerance [58] and other organismal stress responses [59,60]. Additionally, high yeast intake elevates baseline metabolism [30,61], potentially pushing organisms closer to critical metabolic limits due to excessive oxygen demand during heat stress [62].

To manipulate nutritional value, we varied the yeast amount while standardizing other ingredients (including water), which unavoidably altered food density and consistency. Although we could not quantify potential consumption differences due to these physical changes, we infer that adding yeast to food enhanced the nutritional value of consumed food because flies raised on higher-yeast diets in our experiment developed faster and grew larger (personal observation), consistent with observations from other studies [63,64].

### (b) Effects of parasitism on heatwave tolerance

In aphids, the impact of heatwaves on populations can be mitigated when predators or parasitoids are present [14,65]. Heatwaves and parasitism had a similar positive interactive effect in our system. Since *Asobara* wasps could almost always successfully parasitize all available *D. melanogaster* under heatwave regimes used for native hosts (electronic supplementary material, figure S1), we ruled out the possibility in our system that heatwave treatments increase the survival of native hosts to parasitism by directly killing the parasitoid larvae within them. This is the mechanism underlying the positive interaction between parasitism and heatwave documented by Malinski *et al*. [16] and Parker & Kingsolver [17] for *Cotesia congregata* and its caterpillar hosts. We further examined another two potential mechanisms: first, attack by parasitoids could trigger biochemical pathways that increase host heat tolerance (figure 2d). For example, Bahar *et al*. [24] found that parasitoid-infected diamondback moth larvae expressed more heat shock protein in response to high temperatures. Alternatively, if individuals that are sensitive to high temperatures are also more likely to die from parasitoid infection, prior exposure to parasitoids would differentially eliminate more heat-sensitive individuals, raising the mean heat tolerance among survivors (figure 2e). Under the latter scenario, the survival from the ‘parasitism plus heatwave’ treatment combination will never exceed that for the ‘heatwave-only’ treatment. However, figure 4 shows that, on a yeast-rich diet (3 SYL: mass of yeast/total mass = 8%; as used in the laboratory insect diet of [29,42]), a higher absolute number of *Drosophila* larvae survived the heatwave if they were exposed to parasitoids, compared with the no-parasitoid treatment. We therefore excluded the overlapping susceptibility model and highlighted the possibility of shared physiological pathways induced by parasitism.

In contrast to the native hosts, *Drosophila melanogaster* larvae showed increased susceptibility to heat when parasitized by *Asobara,* consistent with our initial expectation. The cost of being parasitized may be higher for hosts exposed to novel parasitoids, increasing vulnerability to the additional stress imposed by heat. However, we could not exclude the possibility that the parasitoid wasps might directly die from the harshest heatwave regime, and hosts also failed to develop due to parasitism interference [66]. Additionally, logistical constraints meant that heatwave treatments for *D. melanogaster* involved no intermediate steps during temperature ramping, potentially triggering different biochemical pathways from those associated with a more gradual temperature rise [24,67]. Therefore, our results in *D. melanogaster* are indicative but not conclusive evidence that infection by novel parasitoids does not offer cross-protection but instead becomes a physiological burden to the non-native hosts in the face of heat stress.

Our experimental design used high parasitism loads to maximize individuals that had interacted with parasitoids, clarifying links between parasitism and heat stress within the host body. However, we must proceed with caution when extrapolating to natural systems, where 100% parasitism rates, although feasible, are rare [39,68,69] and hosts vary in species composition and quality (general ability to survive stressors). If parasitoids actively avoid low-quality hosts, mortalities from parasitism and heatwaves may overlap less, resulting in synergistic effects. Selective targeting [68] could reduce parasitism of unsuitable hosts like *D. bipectinata*, reducing the impact of such cryptic interactions in reality. However, high egg loads and low likelihood to encounter hosts in nature may weaken host-quality assessment [70]. Consequently, egg misplacement into less-suitable hosts may be widespread, even in the presence of other suitable host species [71–73]. Future studies with lower parasitoid-to-host ratios and diverse host species are needed to assess these impacts in natural communities.

### (c) Context dependence of the interactive effect between parasitism and heatwave

The positive interaction between parasitism and heatwave treatments disappeared under low yeast conditions. This may reflect protein deprivation that limits parasitoid-induced response to heat stress or already-elevated baseline levels of stress-responsive molecules (such as trehalose mentioned previously) under very poor nutrition. At high yeast levels, the baseline thermal tolerance is low, and hosts have enough resources to mount strong stress responses when parasitized. Complementary transcriptomic or proteomic analysis of individuals reared at different yeast levels and upon stimulation could test these mechanistic hypotheses.

Lineages with different capacities of thermal responses have been shown to exhibit different levels of cross-protection between immune and thermal challenges [32]. Our experimental design could not reveal such intra-specific variations across large geographic ranges. Instead, we pooled adjacent lineages for each rainforest species to average lineage differences. Across native host species, we demonstrated a consistent pattern of interactive effects despite differences in outcomes of host–parasitoid interactions. However, this cross-protection between parasitism and heatwave treatment may not fully extend to hosts exposed to novel parasitoids, implying that only certain immune pathways facilitate heat tolerance.

Notably, our results provide additional evidence that parasitoids attack hosts unsuitable for offspring development [73–75], and such cryptic interactions have fitness consequences when the hidden hosts face subsequent stressors. In natural systems, female parasitoids may attack up to 80% more host individuals than those yielding successful emergence [76], and molecular methods increasingly reveal the prevalence of parasitoids targeting unsuitable hosts [71,77]. Occurrence of host–parasitoid interactions is likely underestimated [72,78], as are the ecological impacts of failed attacks [19,79]. These non-reproductive interactions could still influence host and parasitoid demography [80] and the subsequent parasitoid interactions with suitable hosts [73,81–83]. Our work extends the non-reproductive effects to include host tolerance to subsequent stress such as extreme heat and underscores the need to study cryptic host–parasitoid interactions to understand population dynamics in the face of multiple stressors.

## 5. Conclusions

Predicting the interactive effects of multiple stressors remains a central challenge in global change ecology. Generalizing these effects hinges on understanding whether stressors rely on common physiological pathways or different pathways that compete for resources. Our study characterizes unexpected relationships between heat tolerance and an organism’s internal states induced by yeast deficiency or parasitoid exposure. Stressful conditions such as yeast deprivation and immune challenges enhanced heat tolerance, suggesting a general cross-protective mechanism among responses to different stressors. If physiological crosstalk is confirmed, interactions with parasitoids could act to stabilize host populations against fluctuations of host populations driven by extreme heat. By applying the heatwave event long after the parasitism attack, we focused on revealing how heat tolerance was altered by parasitism history. Meanwhile, temperature can directly affect parasitoid virulence and host immunity [15,16], further complicating the ultimate outcome of host–parasitoid interactions under multiple stressors. Notably, such non-reproductive effects of parasitism will influence a broader range of species, not just their immediate hosts. We conclude that the process of host–parasitoid interactions, not just the reproductive outcome, and their physiological impacts on both host and parasitoid deserve greater attention when studying community response to ongoing climate change.

## Data Availability

The data and code for analysis that support the findings of this study can be viewed on Figshare [84] Supplementary material is available online [85].
